# Ischemia-reperfusion injury with a model of porcine whole-blood *ex-vivo* lung perfusion

**DOI:** 10.3389/frtra.2025.1651671

**Published:** 2025-09-02

**Authors:** Jean-Baptiste Menager, Julia Mercier, Justin Issard, Maria-Rosa Ghigna, Jeanne Tran Van Nhieu, Benoit Decante, Julien Guihaire, Elie Fadel, Fabrice Antigny, Olaf Mercier

**Affiliations:** ^1^ INSERM UMR_S 999, Pulmonary Hypertension: Pathophysiology and Novel Therapies, Marie Lannelongue Hospital, Le Plessis-Robinson, France; ^2^ School of Medicine, Pulmonary Hypertension: Pathophysiology and Novel Therapies (HPPIT), Paris-Saclay University, Le Kremlin-Bicêtre, France; ^3^ Department of Thoracic Surgery and Heart-Lung Transplantation, Paris-Saclay University, Marie-Lannelongue Hospital, Le Plessis Robinson, France; ^4^ Pathology Department, Gustave Roussy Institute, Villejuif, France; ^5^ IMRB INSERM U955, Paris-Est Créteil University, Créteil, France

**Keywords:** ischemia-reperfusion, animal model, organ perfusion, lung transplant, primary graft dysfunction

## Abstract

**Introduction:**

Our objective was to model Ischemia-Reperfusion (IR) injuries by *ex-vivo* perfusion of porcine lungs with whole blood containing the inflammatory cells.

**Methods:**

Lungs and whole blood were collected from 12 pigs and submitted to cold ischemia time (CIT) of 1 or 18 h. The lungs were then ventilated and perfused for 6 h at 37°C using donor whole blood. Pulmonary pressure was 20 mmHg.

**Results:**

Compared to the short CIT group, the long CIT group had a lower maximum perfusion flow rate (mean difference in % cardiac output, −39%; 95% CI, −66 to −12; *P* = 0.005) and higher pulmonary vascular resistance (mean difference, 1,077 dyne·s·cm^−^⁵; 95% CI, 685–1,469; *P* < 0.001). Neutrophils decreased more in the long CIT group (mean difference, −744.02 cells/mm^3^; 95% CI, −1,343.11 to −144.92; *P* = 0.017), suggesting sequestration in the lung parenchyma. Interleukin-6 and −8 levels after 6 h were significantly higher in the long CIT group (mean differences, 1.1 pg/ml; 95% CI, 0.39–1.8; *P* = 0.003; and 29.31 pg/ml; 95%CI, 16.00–42.61; *P* < 0.001; respectively). Progressive microvasculopathy resulting in lymphangiectasia and peribronchovascular inflammatory infiltrates were seen in both groups.

**Conclusion:**

After 18 h of CIT, *ex-vivo* whole-blood perfusion for 6 h replicated features of IR injuries.

## Introduction

The term ischemia-reperfusion injury (IRI) refers to the set of cellular and biochemical damages caused by the reoxygenation of tissue that has undergone a period of hypoxia, either due to an oxygen deficit or to an interruption of blood perfusion. IRI is a common pathophysiological mechanism in human medicine. It is implicated in a wide range of situations: crush syndrome in trauma, revascularization following cerebral or myocardial infarctions, extracorporeal circulation, and solid organ transplantation (1).

In the field of lung transplantation more specifically, IRI is considered the key mechanism behind the formation of inflammatory edema that sometimes develops in the graft after implantation. Clinically, this manifests as a respiratory distress syndrome known in this context as primary graft dysfunction (PGD) (2).

Despite progress in the overall description of IRI mechanisms, there is currently no specific treatment for this phenomenon in clinical practice. Therefore, it is important to continue studying IRI experimentally to deepen our understanding. Preclinical models of pulmonary IRI exist but none have described, to our knowledge, the characteristics of an isolated lung perfused with whole blood. *Ex vivo* perfusion would offer the advantage of a controlled system and could serve as a platform for testing hypotheses by varying lung perfusion and ventilation conditions. It would also be possible to test the effects of new therapies at a preclinical stage using this model.

Our objective was to develop a model of severe pulmonary IRI and to study the physiological characteristics of porcine lungs perfused *ex vivo* with whole blood after a period of cold ischemia.

## Materials and methods

### Ethics statement

All animals received standard care in accordance with French law for animal research. The protocol was approved by the Marie Lannelongue Hospital institutional review board and by the French committee for animal research (APAFIS). The principles of replacement, refinement, and reduction were followed. This manuscript was written in compliance with the ARRIVE 2.0 guidelines for animal research (3).

### Study design

**Figure 1:**
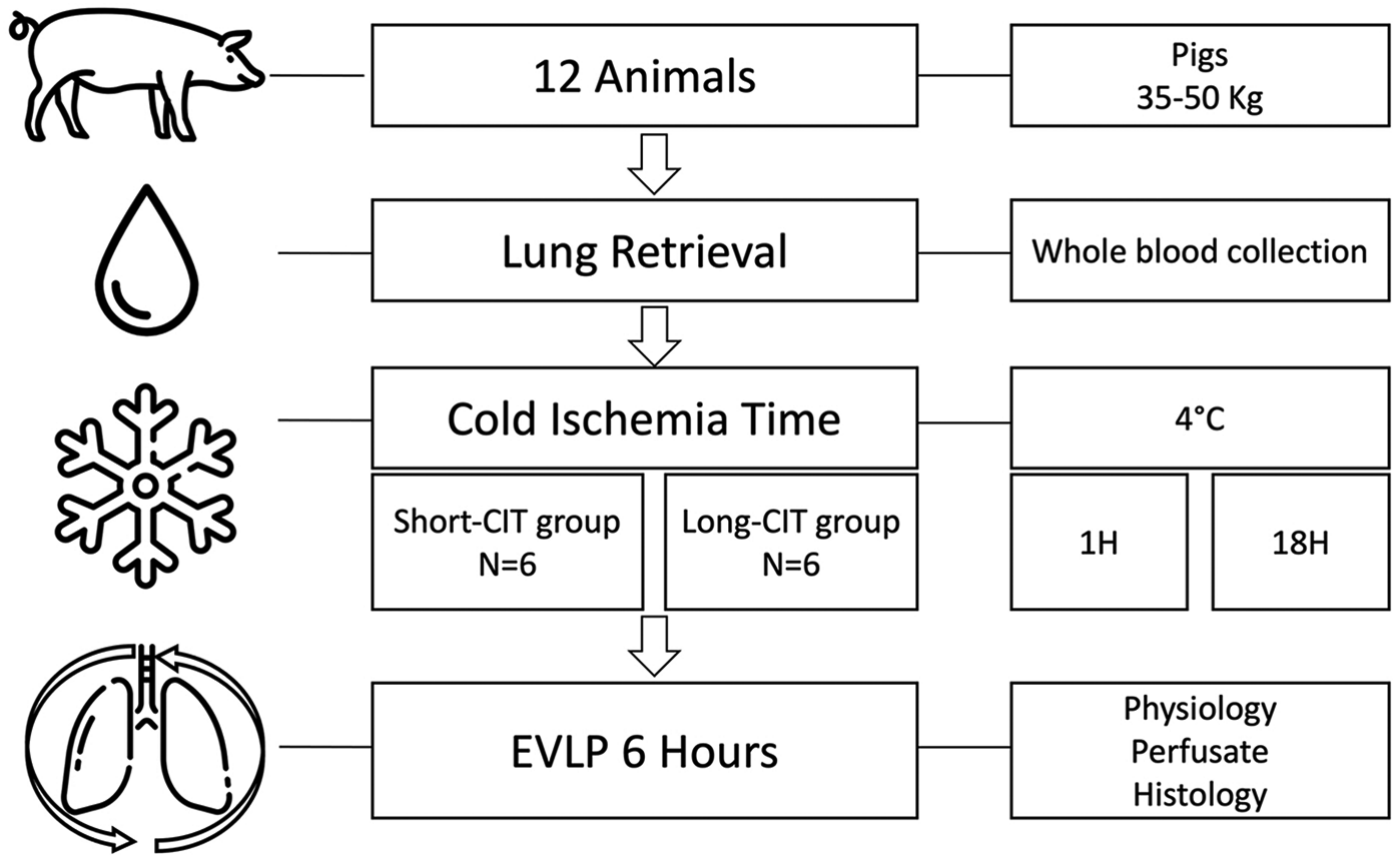
Experimental protocol. Twelve adult pigs underwent lung recovery using the same technique as for human lung procurement. During the procedure, the donor's whole blood was also collected, and both the lungs and blood were stored at 4°C. The animals were randomly assigned to either a short cold ischemia time (S-CIT) of 1 h (6 animals) or a long CIT (L-CIT) of 18 h (6 animals). The lungs were then perfused with the donor's whole blood rewarmed at 37°C and ventilated for 6 h. During perfusion, physiological, biological, and histological changes were assessed.

Figure 1 shows the experimental protocol. The lungs and whole blood were retrieved from 12 Mangaliza pigs. The animals were allocated in a 1:1 ratio to a cold ischemia time (CIT), with static preservation at 4°C, of 1 h or 18 h. The 1-hour period allowed the operator sufficient time to prepare the next phase of the experiment, while the 18-hour period was chosen based on previous reports indicating that it maximizes IRI in pig lungs (4).

*Ex-vivo* lung perfusion (EVLP) with the whole blood of the donor animal was then performed for 6 h in both groups.

### Lung and blood recovery

The pigs were anesthetized with repeated intravenous injection of propofol (3 mg/kg), cisatracurium (0.3 mg/kg), and sufentanil (0.2 mg/kg). A Swan-Ganz catheter was inserted into the pulmonary artery via percutaneous puncture of the superior vena cava. Correct catheter placement was confirmed by radiography. Pulmonary artery pressures and cardiac output (CO) were measured three times, and the mean value was recorded.

**Figure 2:**
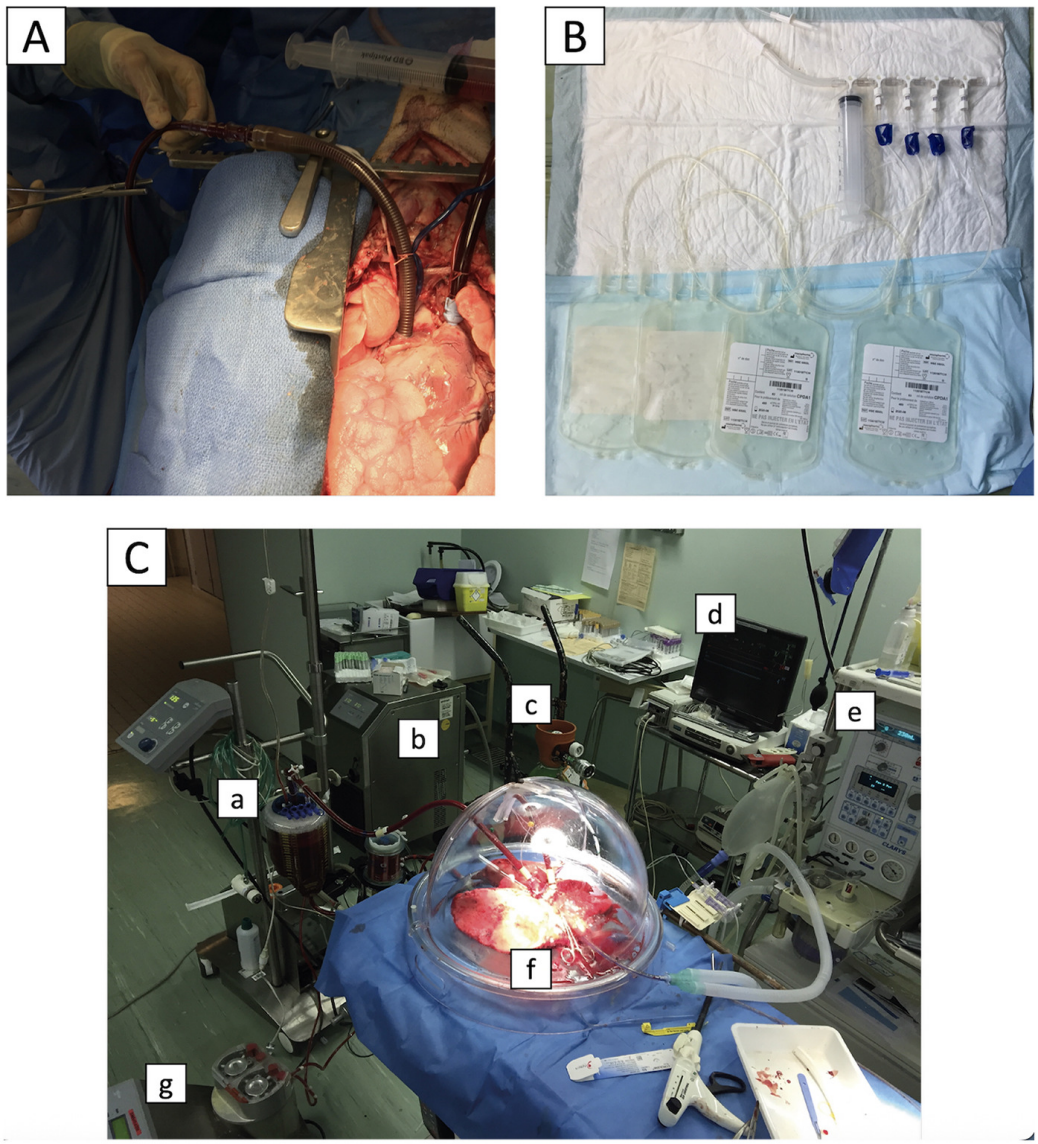
Blood collection and *ex-vivo* lung perfusion techniques. Before pneumoplegia, a cannula was inserted into the inferior vena cava **(A)** and connected to blood collection bags **(B)** The animal was then placed in the Trendelenburg position, and 1.5 L of blood was collected by gravity and stored for subsequent lung perfusion. The *ex-vivo* lung perfusion circuit is shown in **(C)**: pump and blood reservoir (a), thermal heater (b), gas mix for carboxylation (c), pressure monitor (d), mechanical ventilator (e), lungs in an isothermic chamber (f), and recovery line (g).

A median sternotomy was performed. After heparinization (30,000 IU), the pulmonary artery was cannulated and connected to a flushing line. The inferior vena cava was cannulated (Ultraflex 28Fr, Medtronic, Dublin, Ireland) through the right atrium and connected to blood-collection bags (CPDA1 blood storage bag, Macopharma, Mouvaux, France) (Figure 2). The left hemi-azygos vein was ligated. The pig was placed in the Trendelenburg position, the atriocaval cannula clamp was released, and 1.5 L of blood was collected, within less than 5 min. The vena cava was then ligated, and the left atrium was opened.

The lungs were flushed through the pulmonary artery with 2 L of 4°C Perfadex Plus® (XVIVO Perfusion, Mölndal, Sweden) and 1,000 µg of epoprostenol (Flolan®, GSK, London, UK). The lungs were recovered, inflated with 50% FiO_2_, and stored at 4°C in bags after a retrograde pneumoplegia with 1l of Perfadex Plus® for either the short period of 1 h (S-CIT group) or the long period of 18 h (L-CIT group).

### Lung perfusion

**Figure 3:**
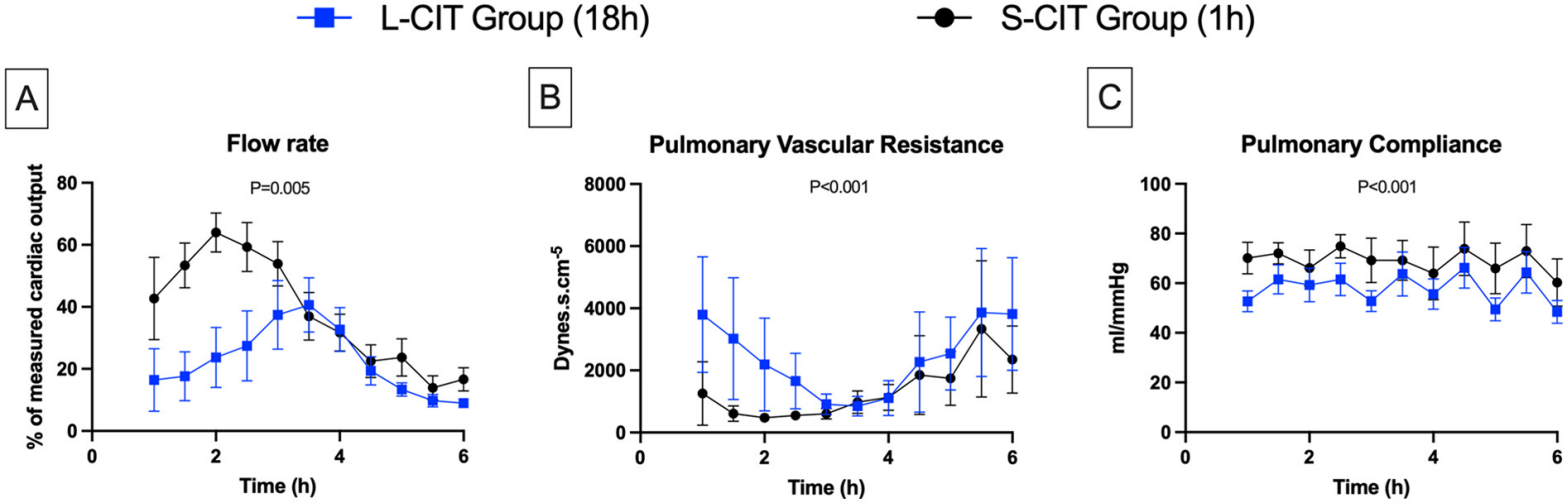
Changes over time in lungs physiological data. **(A)** flow rate, **(B)** pulmonary vascular resistance, **(C)** pulmonary compliance. By maintaining a constant pulmonary arterial pressure of 20 mmHg, the perfusion flow rate could be increased during the first 2–3 h of *ex-vivo* lung perfusion in both groups. However, the maximum flow rate achieved was on average significantly lower in the L-CIT group compared to the S-CIT group, and no organ could be perfused at 100% of the donor's cardiac output. After this initial phase, pulmonary vascular resistance increased significantly, requiring a reduction in the perfusion flow rate to prevent the arterial pressure from exceeding 20 mmHg.

After 1 h or 18 h of cold static preservation, the lungs were connected to a closed circuit (EVLP circuit kit without the leukocyte filter, XVIVO) consisting of a centrifugal pump and a blood reservoir. The left atrium and pulmonary artery were cannulated, and the trachea was intubated. The circuit was primed with donor whole blood and 10,000 IU of heparin (Figure 3).

The perfusion pressures of the pulmonary artery (20 mmHg) and left atrium (5 mmHg) were chosen to simulate the conditions experienced by lung grafts at the time of clamp release in human clinical practice. The perfusion flow rate was continuously measured and constantly adjusted to maintain the pulmonary arterial pressure (PAP) at 20 mmHg. The level of the reservoir was adjusted to maintain the left atrial pressure at 5 mmHg throughout the procedure.

The temperature was gradually increased to 32°C after 20 min of perfusion and maintained at 37.5°C from 30 min onward. Ventilation was started once the graft reached 32°C, with the following parameters: tidal volume, 7 ml/kg/min; positive end-expiratory pressure (PEEP), 5 mmHg; FiO_2_, 21%, and respiratory rate, 7 breaths/min. Every hour, the lungs were challenged by increasing the tidal volume to 10 ml/kg/min, FiO_2_ to 100%, and the respiratory rate to 10 breaths/min, for 10 min, to allow assessments. The gas flow of the EVLP system was initiated with ventilation and adjusted to maintain an inflow PaCO_2_ between 35 and 45 mmHg.

### Lung function assessment

Every 30 min, we recorded the flow rate required to maintain a pulmonary artery pressure of 20 mmHg, as a percentage of the donor's CO. Pulmonary vascular resistance (PVR, dyne·s·cm^−^⁵) was calculated as [(PAP-LAP)/Flow rate] × 80, where PAP is the pulmonary artery pressure and LAP the left atrial pressure.

After one hour then every 30 min, we recorded the ventilatory peak pressure (Ppeak) and mean pressure (Pmean). Pulmonary compliance (ml/mmHg) was calculated as Tidal volume/(Ppeak-PEEP).

Blood gas analyses were performed simultaneously in the inflow and outflow circuits after each lung challenge. Graft oxygenation capacity was expressed as PaO_2_ outflow - PaO_2_ inflow (ΔPaO_2_).

### Laboratory tests for ischemia-reperfusion injury

Hourly, 5 ml of perfusate was taken for analysis and 5 ml of blood was centrifuged at 3,000 rpm for 10 min and the supernatant stored at −80°C for further analysis.

Perfusate levels of interleukin (IL)-2, IL-4, IL-6, IL-8, IL-10, IL-12, IL-18, and TNFα were measured using a Bio-Plex Pro Human Cytokine Assay kit (Bio-Rad Laboratories, Mississauga, Canada), following the manufacturer's protocol. The analytes were read with a Bioplex200 analyzer (Bio-Rad Laboratories), and the data were analyzed using Bio-Plex Manager 6.0 (Bio-Rad Laboratories).

### Histological assessment of ischemia-reperfusion injury

Lung samples from the left lower lobe were taken hourly for a descriptive histological evaluation. After 6 h, lung samples from the left superior lobe were taken for a comparative histological assessment. Specimens were fixed in buffered 4.5% formaldehyde for standard microscopic examination. Sections were colored with hematoxylin eosin saffron. Two slides per animal were assessed by the same lung pathologist who was blinded to data on the animals.

Based on our previous observations, we developed a semi-quantitative descriptive histological score for IR injury, based on four items, each with subcategories: edema (subpleural, septal, peribronchiolar, perivascular), lymphangiectasis (septal, peribronchiolar), inflammatory infiltrate (subpleural, septal, peribronchiolar, perivascular, intra-alveolar) and vascular congestion. Each subcategory was rated 0 (absent), 1 (present, <25% of section), 2 (present, 25%–50% of section), or 3 (present, >50% of section). The score could thus range from 0 (no lesions) to 36 (most severe lesions).

To evaluate lung edema formation, at the end of EVLP, the right superior lobe was dissected, weighed, and heated at 80°C for 7 days to allow calculation of the wet/dry lung weight ratio.

### Statistical analysis

The variables are described as mean ± SD. The Shapiro–Wilk test was applied to assess variable distribution. Comparisons were with Student's *t* test for normally distributed variables and with the non-parametric test Mann–Whitney test for skewed variables. Repeatedly measured variables were evaluated using two-way analysis of variance (ANOVA) and linear regression with or without an interactive term (time or groups) depending on the significance of the interaction verified by the ANOVA. Time was handled as a categorical variable in the regression analyses. The linear regression results were expressed as the estimated mean differences with their 95% confidence intervals (95% CI).

All the statistical analyses were performed with the R program (v4.1.2, open source, http://cran.r-project.org/) and graphs were drawn with Prism® V8.0 (GraphPad, La Jolla, CA). All tests were two-sided and *P* values <0.05 were considered significant.

## Results

### Lung physiology parameters

With a constant pulmonary pressure of 20 mmHg, the perfusion flow rate (Figure 3A) increased over 2 h in the S-CIT group from 42% ± 32% to 64 ± 15% of CO and over 3.5 h in the L-CIT group, but only from 16% ± 24% to 40% ± 21% of CO. During the remainder of the procedure, the flow rate had to be decreased in both groups to prevent an increase in pulmonary pressure. The maximum achievable flow was significantly lower in the L-CIT group during the first 3 h of perfusion (estimated mean difference: −39% of CO; 95% CI, −66 to −12; *P* = 0.005).

Conversely, PVR (Figure 3B) decreased initially (S-CIT: from 1,253 ± 1,021 dyne·s·cm^−^⁵ at 1 h to 478 ± 129 dyne·s·cm^−^⁵ at 2 h: L-CIT: from 3,799 ± 1,862 dyne·s·cm^−^⁵ at 1 h to 848 ± 313 dyne·s·cm^−^⁵ at 3.5 h) then rose sharply. Overall, PVR remained significantly higher in the L-CIT group (estimated mean difference, 1,077 dyne·s·cm^−^⁵; 95%CI, 685–1,469; *P* < 0.001).

Pulmonary compliance (Figure 3C) exhibited a sawtooth pattern in both groups due to the increase in ventilation pressures during the hourly challenges. The range was 60 ± 23–75 ± 11 mmHg in the S-CIT group and 48 ± 11–66 ± 20 mmHg in the L-CIT group. Overall, compliance was significantly lower in the L-CIT group (estimated mean difference, −11.17 mmHg; 95% CI, −17 to −5; *P* < 0.001).

### Blood components

**Figure 4:**
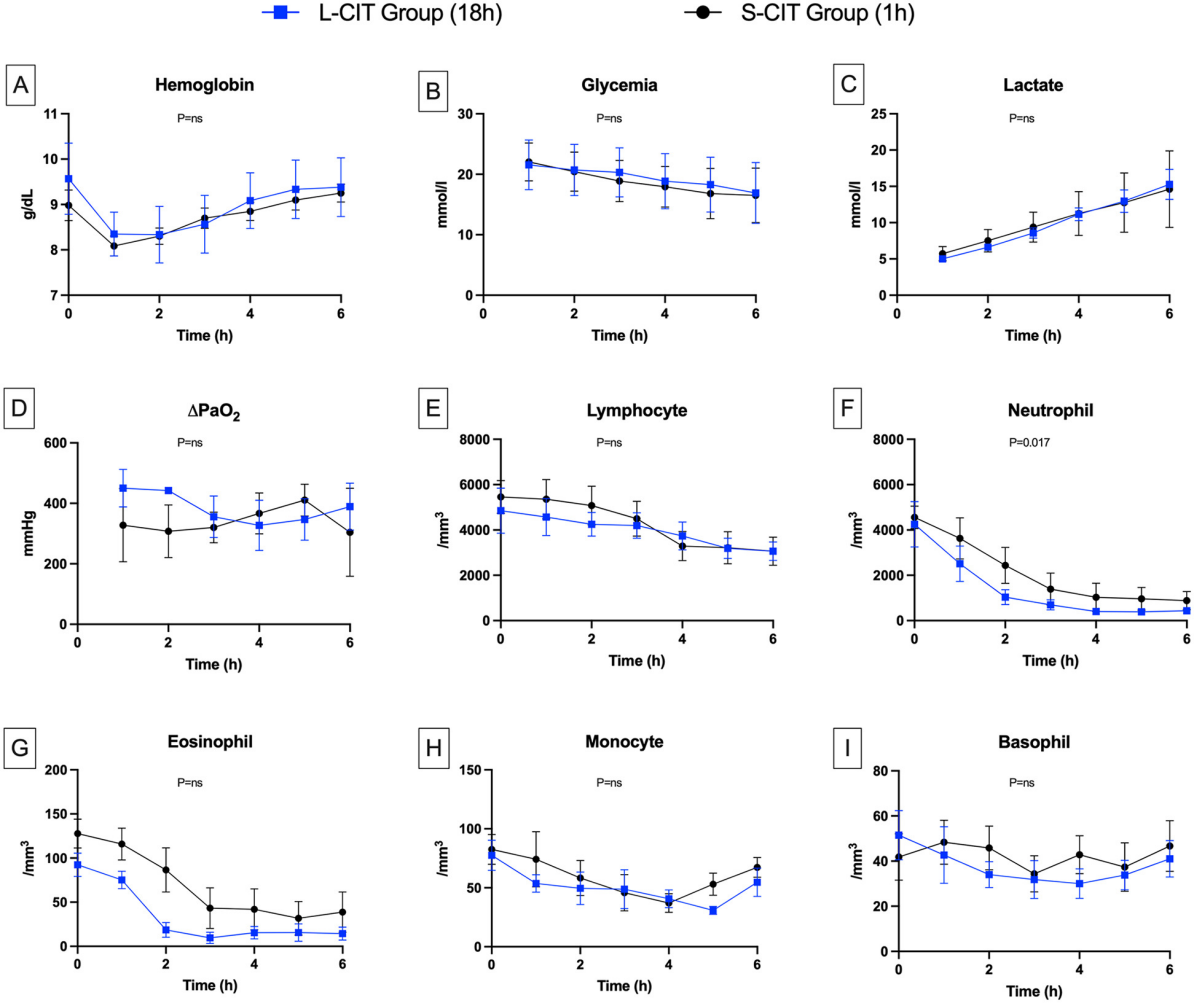
Changes over time in blood components. **(A)** Hemoglobin, **(B)** Glycemia, **(C)** Lactate, **(D)** ∆PaO_2,_ **(E)** Lymphocyte, **(F)** Neutrophil, **(G)** Eosinophil, **(H)** Monocyte, **(I)** Basophil. Delta PaO_2_ remained high and generally stable in both groups. This result is consistent with the low perfusion flow rates, which led to an abnormally long contact time between the blood and the alveolar-capillary membrane. The circulating neutrophil count dropped during the first two hours of perfusion, more markedly in the L-CIT group. The nadir coincided with the point at which pulmonary vascular resistance increased. Given the closed-circuit design, we conclude that neutrophils were sequestered in the lung parenchyma.

After the first hour of perfusion, water evaporation in the closed circuit led to a gradual increase in hemoglobin levels (Figure 4A) (S-CIT: 8 ± 0.5–9.2 ± 0.5; L-CIT: 8.2 ± 0.8–9.3 ± 1; *P* = 0.49).

Rewarming resulted in the resumption of aerobic metabolism in the lungs. Thus, glycemia (Figure 4B) decreased linearly (S-CIT: 22 ± 3–16 ± 4 mmol/L; L-CIT: 21 ± 3–16 ± 4 mmol/L; *P* = 0.48). Lactate levels (Figure 4C) increased steadily over six hours (S-CIT: 5.7 ± 1–14.4 ± 5 mmol/L; L-CIT: 5.4 ± 0.8–14.8 ± 3.5 mmol/L; *P* = 0.51).

ΔPaO₂ (Figure 4D), a marker of lung gas-exchange capacity, remained high in both groups throughout the procedure (S-CIT: 307 ± 87–410 ± 52 mmHg; L-CIT: 327 ± 83–450 ± 61 mmHg; *P* = 0.189).

Blood leukocyte counts decreased gradually during EVLP. Lymphocyte counts (Figure 4E) declined moderately over the 6 h (S-CIT: 5,457 ± 1,614–3,066 ± 1,526; L-CIT: 4,852 ± 2,434–3,062 ± 1,002 cells/mm^3^; *P* = 0.42). The most notable change was in neutrophil counts (Figure 4F) (H0, H3, and H6: S-CIT, 4,559 ± 1,111, 1,388 ± 1,738, and 884 ± 988 cells/mm^3^; L-CIT: 4,252 ± 2,452, 699 ± 549, and 434 ± 283 cells/mm^3^). This decrease was significantly greater in the L-CIT group (estimated mean difference, −744.02 cells/mm^3^; 95% CI, −1,343.11 to −144.92; *P* = 0.017).

Eosinophil counts (Figure 4G) also decreased from H0 to H6 (S-CIT: 127 ± 36–38 ± 56 cells/mm^3^; L-CIT: 92 ± 32–14 ± 18 cells/mm^3^; *P* = 0.32). Monocyte (Figure 4H) counts showed little change throughout EVLP (S-CIT: 82 ± 28–67 ± 20 cells/mm^3^; L-CIT: 77 ± 31–54 ± 29 cells/mm^3^; *P* = 0.18). Basophil counts (Figure 4I) from H0 to H6 also remained fairly stable (S-CIT: 41 ± 22–46 ± 26 cells/mm^3^; L-CIT: 51 ± 26–41 ± 19 cells/mm^3^; *P* = 0.32).

### Blood cytokines

**Figure 5:**
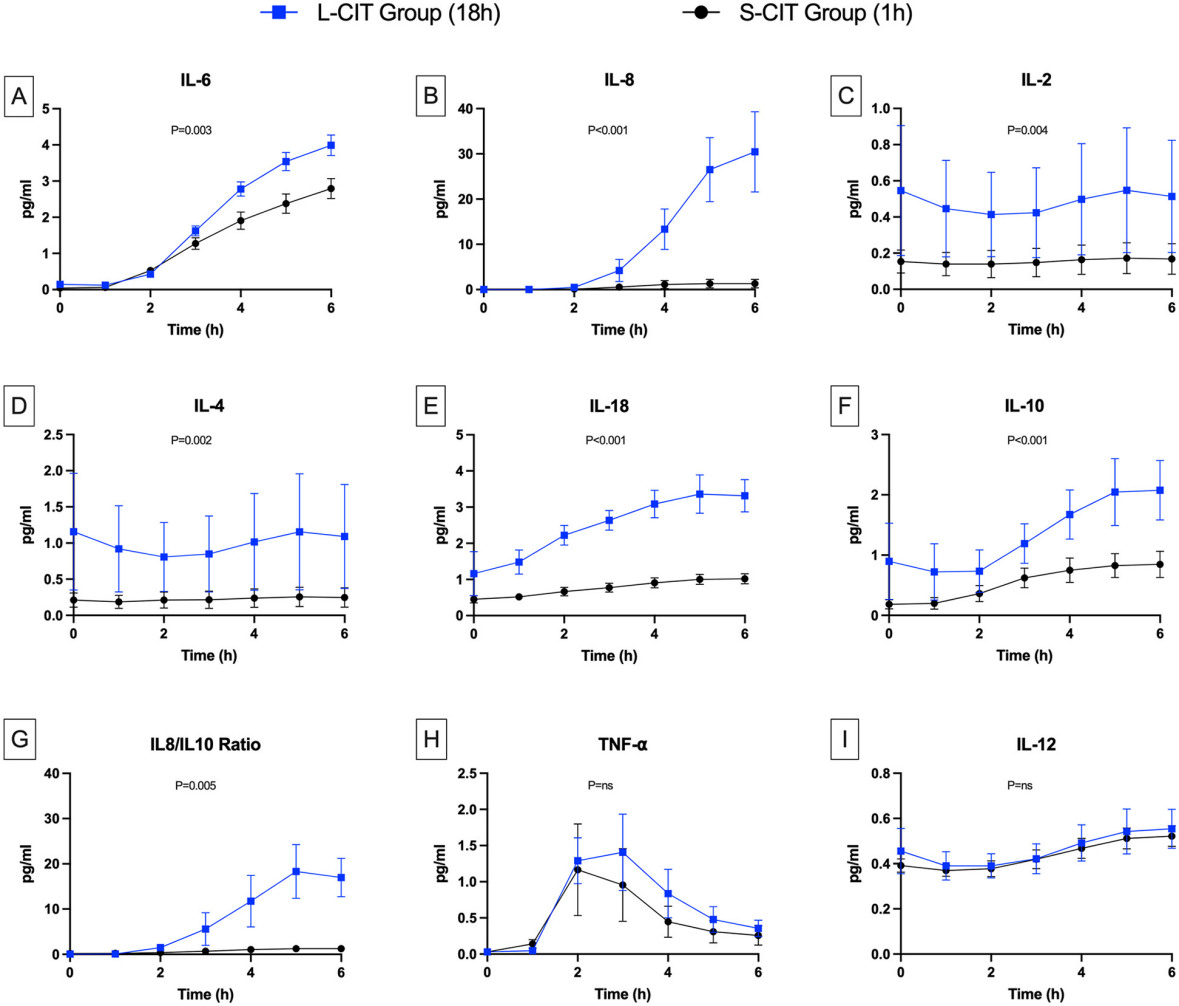
Changes over time in cytokine levels. **(A)** Interleukine 6, **(B)** IL-8, **(C)** IL-2, **(D)** IL-4, **(E)** IL-18, **(F)** IL-10, **(G)** IL8/IL10 ratio, **(H)** TNF-α, **(I)** IL-12. The levels of the pro-inflammatory cytokines IL-6, IL-8, and IL-18 were higher during perfusion in the L-CIT group than in the S-CIT group. Also, the blood concentrations of interleukins IL-10, IL-2, and IL-4 were higher from the start of the perfusion in the L-CIT group, consistent with the longer cold-storage time of the blood in this group.

IL-6 levels (Figure 5A) were similar at baseline between groups and increased significantly after the first two hours of perfusion in both groups (S-CIT: 0.53 ± 0.15–2.79 ± 0.62 pg/ml; L-CIT: 0.48 ± 0.15–3.39 ± 0.86 pg/ml). The increase was significantly greater in the L-CIT group (estimated mean difference, 1.1 pg/ml; 95% CI, 0.39–1.8; *P* = 0.003). IL-8 concentrations (Figure 5B) increased more markedly than IL-6 (S-CIT: 0.11 ± 0.16–1.32 ± 2.10 pg/ml; L-CIT: 0.49 ± 0.55–30.62 ± 20.04 pg/ml; estimated mean difference, 29.31 pg/ml; 95% CI, 16.00–42.61; *P* < 0.001).

IL-2 (Figure 5C) and IL-4 (Figure 5D) levels were significantly higher from H0 to H6 in the L-CIT group (IL-2: 0.55 ± 0.8 and 0.51 ± 0.69 pg/ml; IL-4: 1.16 ± 1.8 and 1.09 ± 1.61 pg/ml) than in the S-CIT group (IL-2: 0.15 ± 0.14 and 0.17 ± 0.19 pg/ml; IL-4: 0.21 ± 0.22 and 0.25 ± 0.3 pg/ml). The estimated mean difference for IL-2 was 0.33 pg/ml (95% CI, 0.11–0.55; *P* = 0.004). Corresponding values for IL-4 were 0.78 pg/ml (95% CI, 0.3–1.25; *P* = 0.002). IL-18 concentrations (Figure 5E) also increased more in the L-CIT group (from 1.16 ± 1.36 to 3.32 ± 1.00 pg/ml) than in the S-CIT group (from 0.45 ± 0.23 to 1.02 ± 0.31 pg/ml), with an estimated mean difference of 1.7 pg/ml (95% CI, 1.36–2.05; *P* < 0.001).

Levels of the anti-inflammatory cytokine IL-10 (Figure 5F) were significantly higher in the L-CIT group, increasing from 0.9 ± 1.42 to 2.08 ± 1.1 pg/ml vs. 0.18 ± 0.17 to 0.85 ± 0.49 pg/ml in the S-CIT group (estimated mean difference, 0.79 pg/ml; 95% CI, 0.43–1.15; *P* < 0.001). Nonetheless, the IL-8/IL-10 ratio (Figure 5G) indicated significantly worse inflammation in the L-CIT group at H6 (estimated mean difference, 15.95 pg/ml; 95% CI, 5.36–26.53; *P* = 0.005). TNF-α concentrations (Figure 5H) followed a different pattern compared to other cytokines, increasing until H3 (to 0.76 ± 0.97 and 1.41 ± 1.18 pg/ml in the S-CIT and L-CIT groups, respectively) then decreasing, with no significant between-group difference (*P* = 0.083). IL-12 concentrations (Figure 5I) were similar in both groups (at H0 and H6: S-CIT group, 0.37 ± 0.06 and 0.52 ± 0.1 pg/ml; L-CIT group: 0.39 ± 0.14 and 0.55 ± 0.19 pg/ml; *P* = 0.42).

### Histology

**Figure 6:**
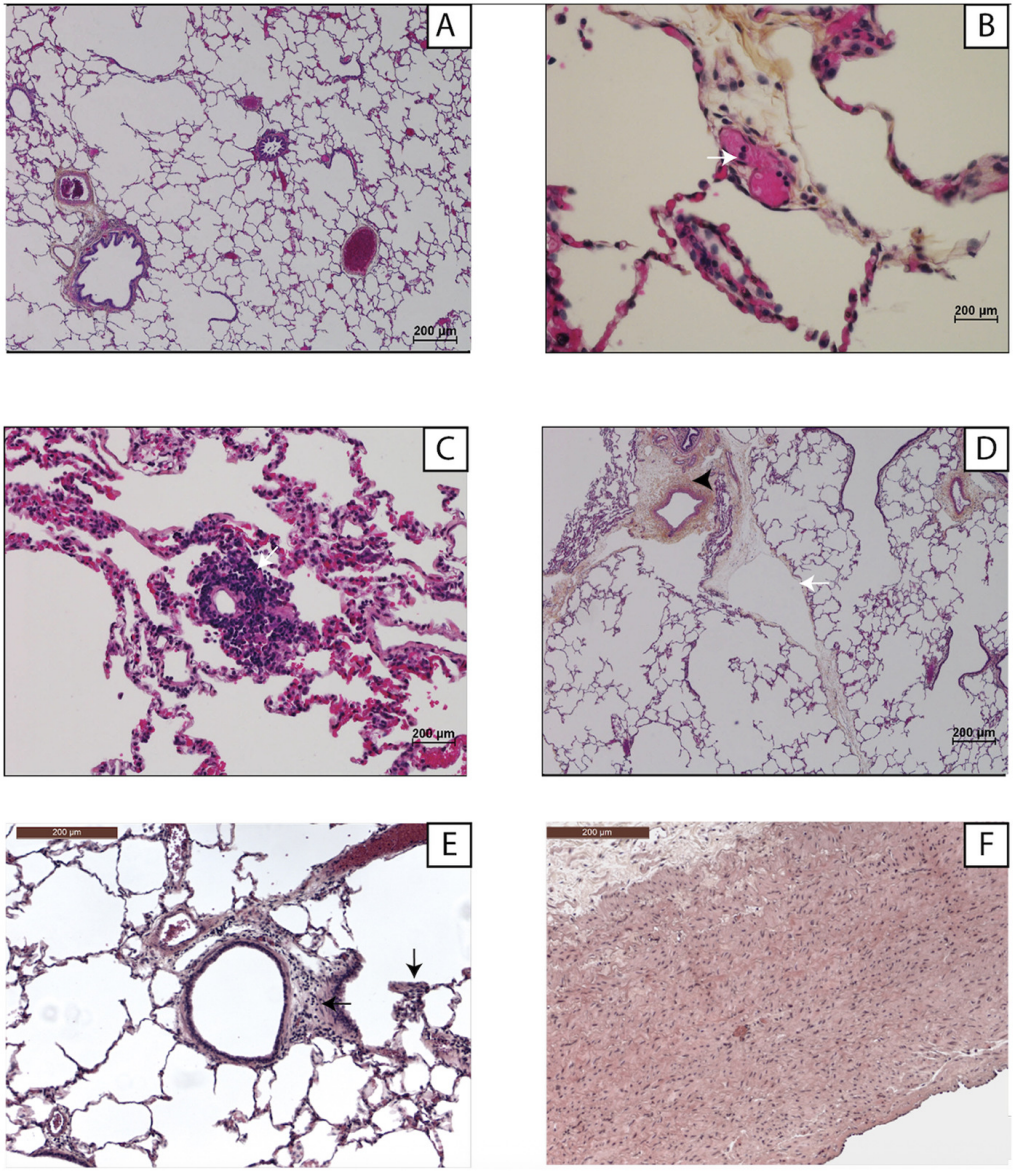
Examples of lung histology findings. Light microscopy with hematoxylin-eosin-saffron staining and ×1,000 magnification. **(A)** Normal lung parenchyma at H0 of *ex-vivo* lung perfusion. **(B)** Neutrophil adhesion (white arrow) to an endothelial cell (H3). **(C)** Neutrophilic capillaritis (H6). **(D)** Perivascular interstitial edema (arrowhead) and lymphangiectasia (white arrow) (H6). **(E)** Interstitial inflammatory infiltrate (black arrow) (H6). **(F)** The large vessels (pulmonary artery trunk) were normal, the endothelial damage being exclusively microvascular (H6).

Figure 6 shows the histological findings. At baseline, the lung parenchyma was normal in both groups. During EVLP, microvasculopathy developed gradually, with leukocyte adhesion to the endothelium initially then progressively worsening neutrophilic capillaritis. These lesions did not affect the large vessels. After 3 h, tissue infiltrates, edema, and lymphatic-vessel dilation developed.

**Figure 7:**
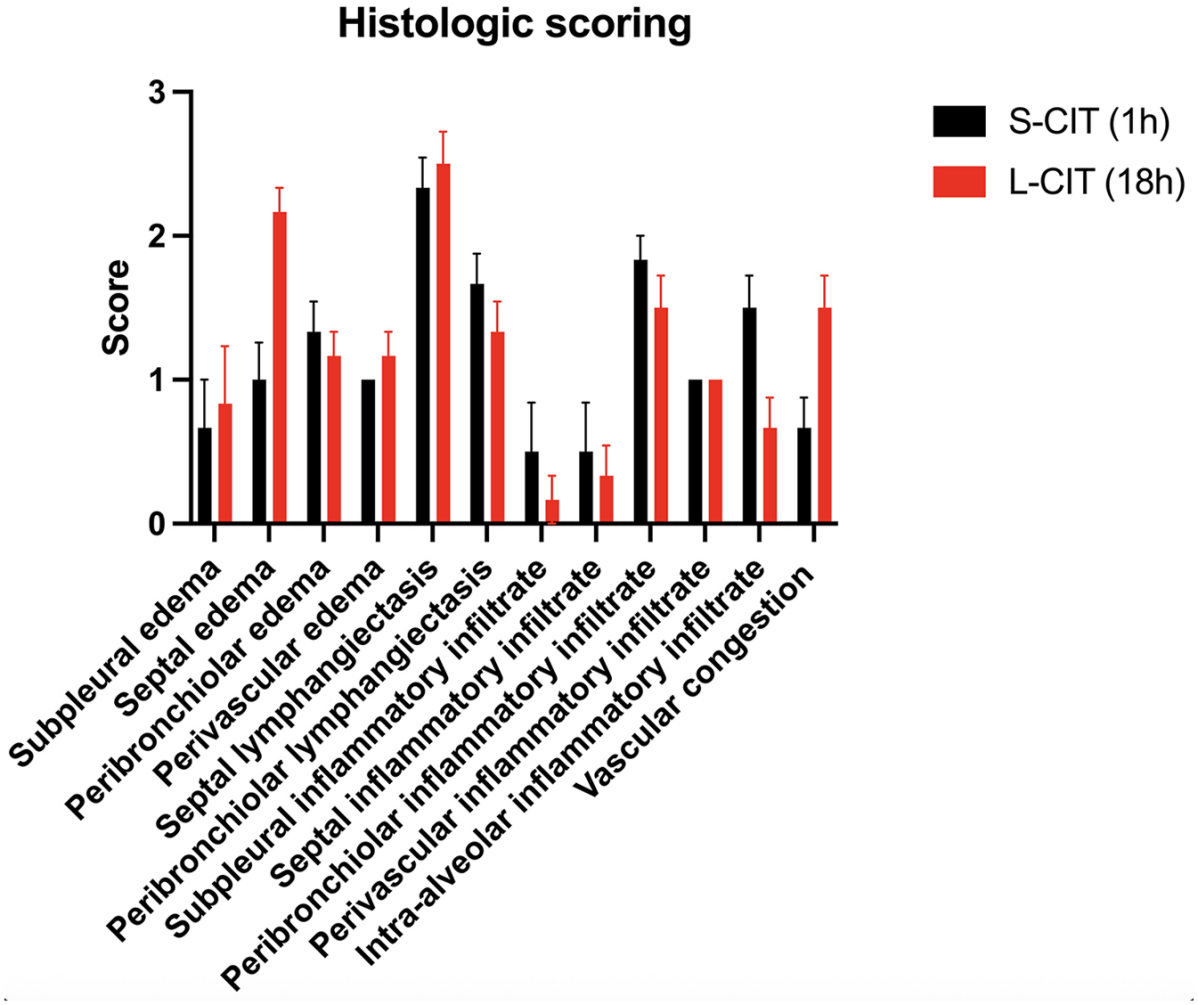
Histology lesion scores in the two groups. The overall score was not significantly different between groups.

At H6 (Figure 7), the most prevalent lesions in the S-CIT group were interseptal lymphangiectasia (mean score, 2.3 ± 0.2), peribronchiolar infiltrates (1.8 ± 0.2), and peribronchiolar lymphangiectasia (1.7 ± 0.2). The most severe lesions in the L-CIT group were interseptal lymphangiectasia (2.5 ± 0.2), septal edema (2.2 ± 0.2), and vascular congestion (1.5 ± 0.2). The total histological score was not significantly different between groups (S-CIT: 14 ± 1.1; L-CIT: 14.3 ± 0.; *P* = 0.79). The wet/dry ratio was similarly elevated in both groups (S-CIT: 4.9 ± 0.19; L-CIT: 5.4 ± 0.22, *P* = 0.13).

## Discussion

Our main finding is that warm *ex-vivo* perfusion of a porcine lung with whole blood induced inflammatory edema, whose severity was modulated by the duration of cold ischemia. The consequences of the interaction between blood and lungs were adhesion of circulating neutrophils to the pulmonary endothelium, followed by neutrophil infiltration and sequestration in the parenchyma, resulting in microvasculopathy responsible for a PVR increase.

We used an 18 h CIT to enhance the occurrence of IRI. Nevertheless, lesions were also present with a CIT of only 1 h. The changes in physiological and laboratory variables occurred in similar directions in both groups but were more pronounced in the L-CIT group. Several factors may contribute to lesion development even after a short CIT. The static preservation of the blood used for perfusion may have induced neutrophil activation, consistent with the between-group difference in IL-2 and IL-4 levels at H0. Assays of neutrophil degranulation products such as neutrophilic extracellular traps and myeloperoxidase would be of interest. Nevertheless, the blood bags were of a type widely used for collecting human whole-blood donations and contained citrate phosphate dextrose adenine-1, which helps to maintain blood homeostasis, with few changes as storage time increases (5). Whole blood preserved in these bags can be safely transfused to humans with no prior processing (6). Therefore, the effect of the blood storage method on IRI was limited.

The histological damage noted at the end of EVLP was broadly similar in the two groups. To our knowledge, there is currently no gold standard histological scoring system for ischemia-reperfusion injuries. We initially intended to use a semi-quantitative score previously developed by our team (7), which assessed inflammatory infiltrates and tissue edema. However, that system was designed for a protocol without *ex vivo* perfusion. Following a review of preliminary samples from lungs perfused with whole blood, and based on the recommendations of our pathologists, we revised the scoring method to more accurately reflect key features of primary graft dysfunction and to better align with our experimental model. It would be valuable for other researchers to apply this revised score to validate its relevance and assess whether similar histological patterns—namely, edema predominantly located in the interlobular septa and marked peribronchiolar inflammatory infiltrates at the end of perfusion—are consistently observed.

Our results shows that a healthy lung, which was by definition perfused with whole blood and full cardiac output while *in vivo* in the donor, can no longer tolerate full cardiac output when artificially perfused *ex vivo*, even after a negligible ischemia period. The macroscopic-scale and hemodynamic study of the impact of IRI is original. With constant-pressure perfusion, the lung initially became progressively less resistant during 2–3 h. In a second phase, vascular resistance increased sharply, forcing us to reduce the perfusion flow to avoid an excessive rise in PAP. It would be interesting, in future studies, to understand precisely what occurs at the peak of perfusion and what mechanisms explain the shift, synchronous in both groups, from a progressively less resistant to a more resistant profile. One likely explanation is a saturation effect of the pulmonary vascular bed, as we observed severe obstructive capillaritis and septal swelling after 6 h of perfusion, while it is known that endothelial dysfunction leads to increased filtration of fluid into the interstitium and plugging of capillaries by activated leukocyte (8). It would also be interesting to determine whether a potential treatment for ischemia-reperfusion could modify this hemodynamic profile, for instance, by increasing or rightshifting this perfusion peak during the procedure.

Lung ischemia-reperfusion injury has been reported in various conditions where pulmonary blood flow is impaired and restored, such as acute respiratory distress syndrome, cardiopulmonary bypass, pulmonary embolism thrombolysis, and transplantation. In all these scenarios, neutrophil activation and recruitment are considered central mechanisms of IRI (9). A key strength of our model is its ability to replicate this inflammatory response. Cells trafficking into sites of inflammation is driven by cytokines; however, secretion profiles varied depending on the molecule. Concentrations of IL-6, IL-8, and IL-18 progressively increased over time and were significantly higher in the L-CIT group. These findings correlate with De Perrot and al (10) who demonstrated that elevated blood IL-8 level two hours post-transplant is associated with a higher risk of PGD, and that IL-18 levels rise with longer ischemia times. IL-10 levels were also higher in the L-CIT group, despite its known anti-inflammatory properties (11). We interpret this as possible regulatory feedback loop between pro- and anti-inflammatory responses, particularly since the IL-8/IL-10 ratio remained elevated. Interestingly, TNF-α levels showed a similar transient increase in both groups. TNF-α has a key role in early inflammatory responses by upregulating other cytokines (12). Although monocytes are its primary source, TNF-α can also be secreted by lymphocytes, neutrophils, and endothelial cells (13). The subsequent decrease we observed may reflect a saturation effect in these activated cell lines. Moreover, the secretion of IL-6, IL-8, and IL-10 appeared to be uncoupled from that of TNF-α after three hours of perfusion. This may suggest autonomous regulation of these cytokines or the existence of alternative stimulation pathways independent of TNF-α. While we couldn't explore these mechanisms here, they may deserve dedicated future studies. Finally, IL-12 levels remained unchanged in both groups. This may be explained by the absence of alloreactivity in our model, as IL-12 is known to play a role in allospecific cytotoxic responses (14).

Other models of IR lung injury have been reported. *in vitro* models typically involve exposing a cell line of interest to hypoxia followed by reoxygenation in a controlled culture medium (15). This technique is simple and cost-effective, allows for repeated experiments, and provides detailed information on cellular mechanisms. However, *in vitro* studies cannot replicate the complex physiological interactions among diverse cell types in a whole organ. Another model is the hilar clamping technique. The pulmonary hilum of the animal is exposed surgically and clamped to subject the lung to transient warm ischemia (16). After clamp release, the lung is perfused, allowing for analyses to be conducted directly in the parenchyma and in arterial and venous blood. This technique is simple and effective, but it does not include a cold ischemia phase for the lung. It is therefore less relevant for studying IRI in the context of transplantation. *Ex vivo* lung perfusion models have been described in pigs, rats, and even mice (17), primarily with the aim of preserving and optimizing lung grafts. For this purpose, hyperosmotic perfusates, either acellular, such as Steen Solution (18), or cellular, containing red blood cells (19), have been widely used combined with leukocyte depletion to protect the vascular endothelium and prevent edema. In contrast, our model was specifically designed to elicit a robust inflammatory response; therefore, we deliberately chose to use whole blood and excluded any leukocyte-depleting filters from the perfusion circuit. Compared to these previous models, ours may offer an interesting compromise between simplicity and versatility. The ability to analyze lungs over several hours by performing biopsies and blood assays, while controlling and modifying variables such as temperature, flow, pressure and ventilation parameters allows a vast array of experiments and is valuable for dynamic investigations of pure IR mechanisms. Among the potential sophistication of the model, one could consider perfusion with heterologous blood to analyze the allogeneic component, implantation of the lung after perfusion to assess functional outcomes, or the use of human lungs to improve the translatability of the results.

One limitation of our study is that, surprisingly in both groups, PaO_2_ showed little change during EVLP. The most likely reason is the constant perfusion pressure, which required low perfusion flow rates. The resulting long contact time between the blood and the alveolar-capillary membrane may have resulted in artificially high PaO_2_ values. To assess this hypothesis, we performed additional experiments involving an increase in the perfusion rate after 6 h. The effects were substantial pulmonary edema, a drop in blood reservoir volume, and a collapse in gas exchange (data not shown). This model cannot be considered a full representation of primary graft dysfunction (PGD), as it lacks key diagnostic criteria essential for defining the syndrome—namely, radiographic infiltrates and a decrease in the PaO₂/FiO₂ ratio. Furthermore, the use of autologous blood omits a critical component of PGD pathophysiology: the recipient's allogeneic immune response. Finally, the use of *ex-vivo* lungs eliminates the systemic effects that occur with *in vivo* lungs. This model does not replicate the potential role in IR response of renal and hepatic clearance and of non-blood sources of immune cells such as the secondary lymphoid organs.

## Conclusion

In conclusion, the use of warm whole blood in pig *ex vivo* lung perfusion reproduces key features of ischemia-reperfusion injury. This approach allows for the observation of circulating cell recruitment, cytokine release, and lung tissue damage. The *ex vivo* perfusion setting also provides the flexibility to manipulate numerous parameters, enabling a wide range of experiments to dynamically assess pathophysiological changes in the lung. Although further validation is needed, this model could serve as a valuable platform for basic research into ischemia-reperfusion mechanisms and for the preclinical testing of novel therapeutic strategies.

## Data Availability

The raw data supporting the conclusions of this article will be made available by the authors, without undue reservation.
