# Supplementary material for: Ischemia-reperfusion injury with a model of porcine whole-blood ex-vivo lung perfusion
Source: Front Transplant. 2025 Sep 2;4:1651671. doi: 10.3389/frtra.2025.1651671 (PMC12436443; doi:10.3389/frtra.2025.1651671)
Supplement: Supplementary file 1 [file Presentation1.pptx]

## Slide 1
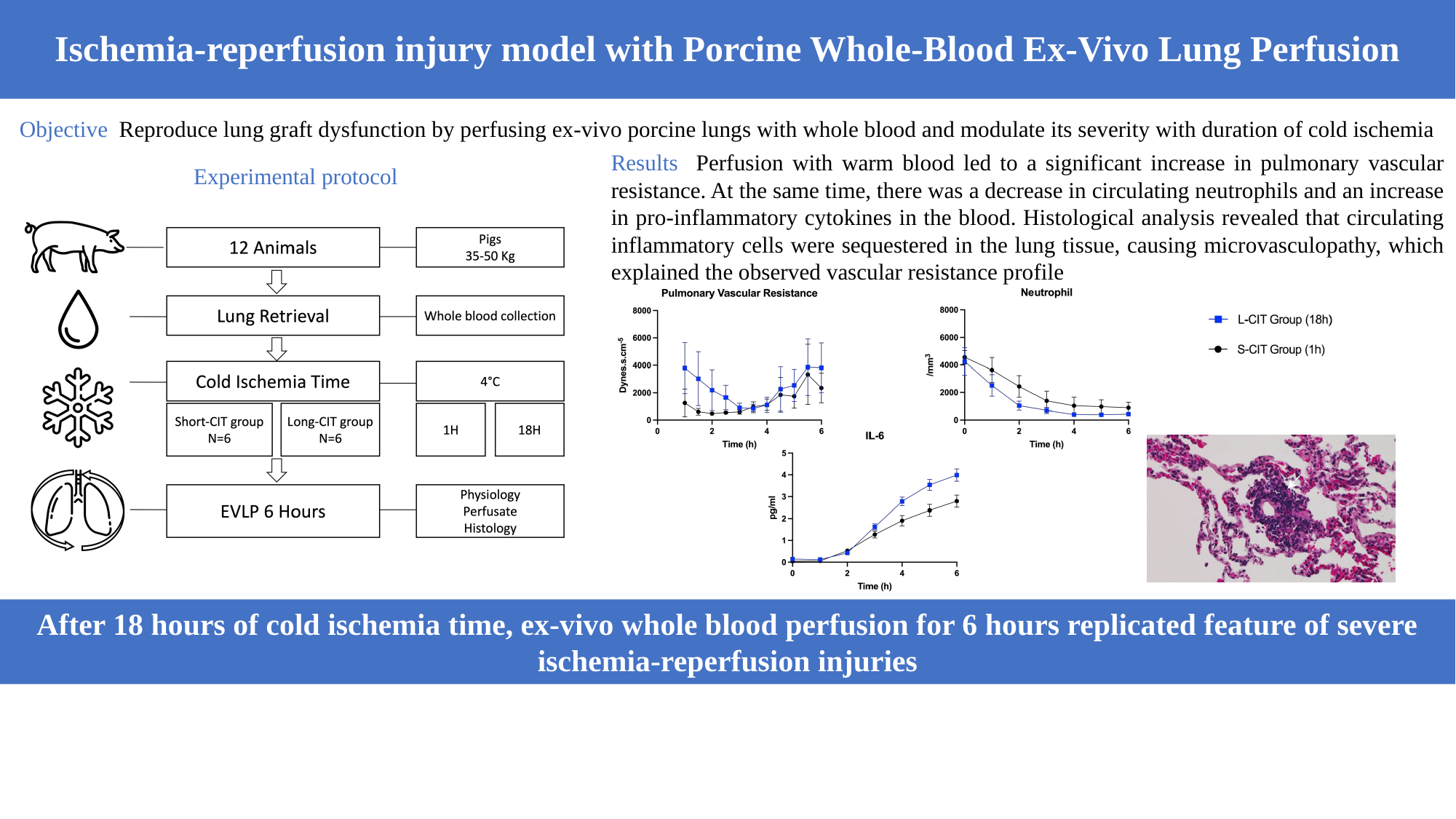

Ischemia-reperfusion injury model with Porcine Whole-Blood Ex-Vivo Lung Perfusion
Objective Reproduce lung graft dysfunction by perfusing ex-vivo porcine lungs with whole blood and modulate its severity with duration of cold ischemia
Results Perfusion with warm blood led to a significant increase in pulmonary vascular resistance. At the same time, there was a decrease in circulating neutrophils and an increase in pro-inflammatory cytokines in the blood. Histological analysis revealed that circulating inflammatory cells were sequestered in the lung tissue, causing microvasculopathy, which explained the observed vascular resistance profile
Experimental protocol
After 18 hours of cold ischemia time, ex-vivo whole blood perfusion for 6 hours replicated feature of severe ischemia-reperfusion injuries
